# Special correlation between diet and MASLD: positive or negative?

**DOI:** 10.1186/s13578-025-01382-1

**Published:** 2025-04-12

**Authors:** Jia Liu, Changmeng Li, Yun Yang, Jingtao Li, Xiaoguang Sun, Yinqiang Zhang, Runping Liu, Fafeng Chen, Xiaojiaoyang Li

**Affiliations:** 1 School of Life Sciences, Beijing University of Chinese Medicine, Beijing, 100029 China; 2 School of Chinese Materia Medica, Beijing University of Chinese Medicine, Beijing, 100029 China; 3 Departments of Infectious Disease, The Affiliated Hospital of Shaanxi University of Chinese Medicine, Xianyang, 712000 China; 4 School of Traditional Chinese Medicine, Beijing University of Chinese Medicine, Beijing, 100029 China; 5 Xiyuan Hospital, China Academy of Chinese Medical Sciences, Beijing, 100091 China

**Keywords:** MASLD, Dietary models, Pathological mechanism, Dietary therapy, Curative strategy, Hepato-intestinal axis

## Abstract

Metabolic dysfunction-associated steatotic liver disease (MASLD) is a chronic and systemic metabolic liver disease characterized by the presence of hepatic steatosis and at least one cardiometabolic risk factor (CMRF). The pathogenesis of MASLD involves multiple mechanisms, including lipid metabolism disorders, insulin resistance, inflammatory responses, and the hepato-intestinal axis of metabolic dysfunction. Among these factors, diet serves as both an inducement and a potential remedy in the disease's development. Notably, a high-lipid diet exacerbates fat accumulation, oxidative stress, and inflammatory responses, thereby promoting the progression of MASLD. Consequently, dietary induction models have become vital tools for studying the pathological mechanisms of MASLD, providing a foundation for identifying potential therapeutic targets. Additionally, we summarize the therapeutic effects of dietary optimization on MASLD and elucidate the role of specific dietary components in regulating the hepato-intestinal axis, lipid metabolism, and inhibiting inflammatory responses. In conclusion, studies utilizing animal models of MASLD offer significant insights into dietary therapy, particularly concerning the regulation of lipid metabolism-related and hepatoenteric axis-related signaling pathways as well as the beneficial mechanism of probiotics in hepatoenteric regulation. By understanding the specific mechanisms by which different dietary patterns affect MASLD, we can assess the clinical applicability of current dietary strategies and provide new directions for research and treatment aimed at disease modification.

**Graphical Abstract:**

The double-edged sword role of dietary intake in the development of MASLD. An unhealthy diet leads to hepatic steatosis, fat accumulation, oxidative stress, and inflammation. In contrast, a balanced diet can prevent or alleviate MASLD progression.
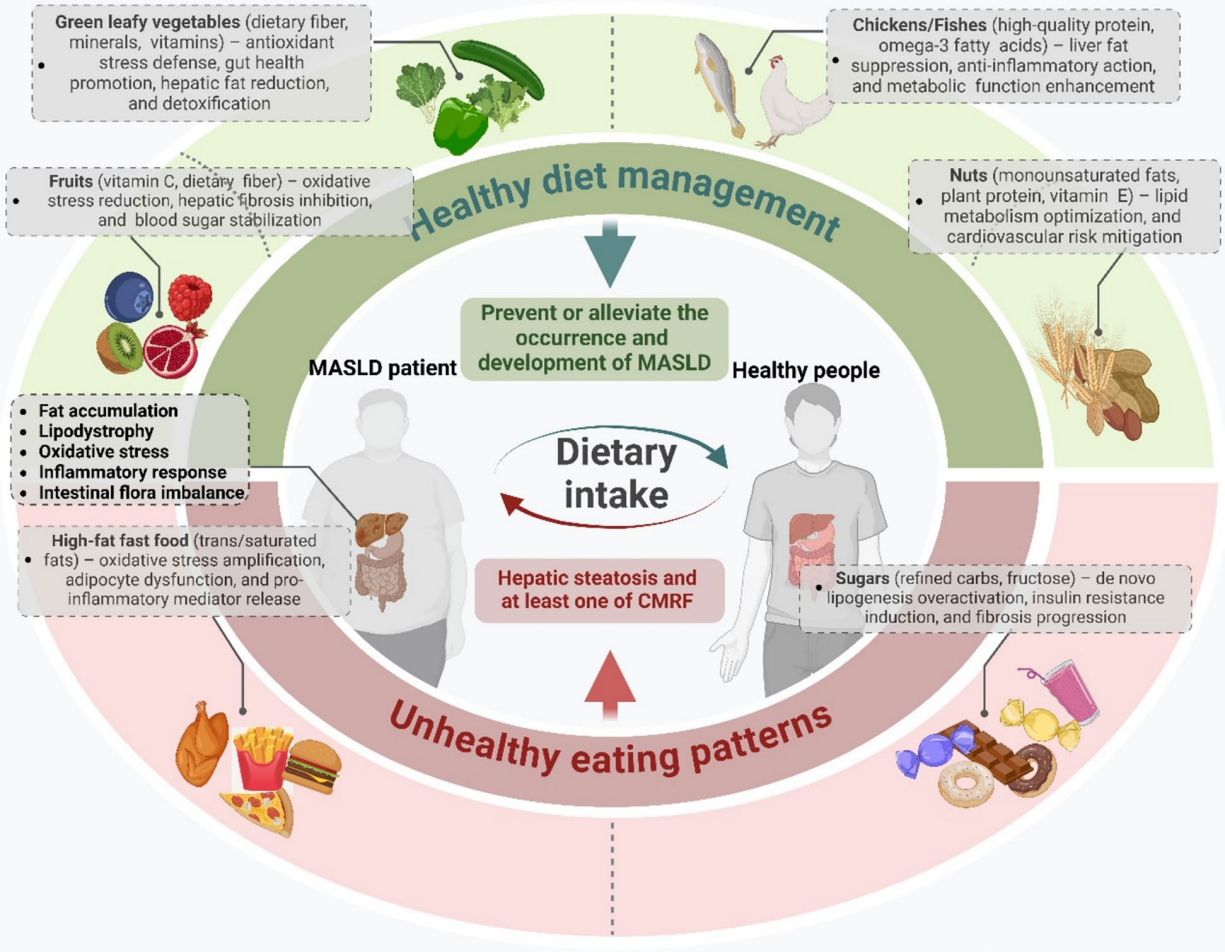

## Introduction

Metabolic dysfunction-associated steatotic liver disease (MASLD) is a newly introduced term that succeeds the previous nomenclatures of non-alcoholic fatty liver disease (NAFLD) and metabolic dysfunction-associated fatty liver disease (MAFLD) that encompasses a broad spectrum of liver diseases linked to metabolic disorders, emphasizing the metabolic abnormalities inherent in fatty liver diseases [1]. MASLD is known to be the most common cause of chronic liver disease, can progress to fibrosis or cirrhosis if left untreated. Notably, the disease is currently sweeping the world and is more frequent in patients with metabolic disorders such as obesity, type 2 diabetes, or metabolic syndromes. Recent in-depth research on MASLD, coupled with an evolving understanding of this disease, has led to a consensus among experts and scholars across various professional fields. A multi-society Delphi consensus statement announced that the diagnostic criteria for MASLD should be based on affirmative criteria rather than exclusion criteria, so that researchers can identify MASLD in the context of regional/ethnic differences and further improve the diagnosis and therapeutic development of this condition. Thus, the new diagnostic condition for MASLD included the presence of hepatic steatosis as well as at least one of the five cardiac metabolic risk factors (CMRF) of metabolic syndromes, but only in the absence of other causes of hepatic steatosis. In particular, CMRF is intended to identify patients for whom insulin resistance (IR) may be the principal underlying factor contributing to hepatic steatosis [2].

## Pathological mechanism of MASLD

MASLD is characterized by inflammation, metabolic disorders, and intestinal flora disorders resulting from hepatic steatosis caused by accumulation of adipose tissue originating in the gastrointestinal tract and within hepatocytes, may even progress to liver fibrosis or even liver cancer. As far as research is concerned, the fats accumulated in livers can be attributed to diets added sugar and rich in saturated, then increasing excessive lipid accumulation [2]. It may lead to the disturbance of lipolysis and adipose de novo synthesis (DNL) function, directly affect the catabolism of triglycerides (TG) and cholesterol, which has a negative effect on fatty acid (FA) oxidation, and further inducing steatosis and lipotoxicity [3]. Furthermore, the liver is not only the central organ of human fat metabolism, but also an important place for carbohydrate metabolism. It is indisputable that excessive co-intake of high fat and high sugar still might contribute to IR, and bring about the functional abnormalities of glucose metabolic pathways such as glycolysis and tricarboxylic acid cycle. Attentively, IR and steatohepatitis are mutually reinforcing phenomena, IR leads to elevated blood sugar levels and fatty acid accumulation, which in turn results in increased blood lipids in the body and even excessive fat deposits in the liver [4]. Subsequently, excessive lipid accumulation disrupts the normal function of liver cells, triggering endoplasmic reticulum stress (ER stress), mitochondrial dysfunction, and production of reactive oxygen species (ROS), which activate crucial signaling pathways and induce inflammatory responses such as immune cell infiltration and apoptosis [5]. These became major drivers of the development of metabolic dysfunction-associated steatohepatitis (MASH).

Although we have confirmed the diagnostic criteria and clinical manifestations of MASLD, there is no efficacious pharmacological agents to combat MASLD in clinical practice. Researchers are making unremitting efforts to explore the pathological mechanism of MASLD, hoping to overcome the disease as swiftly as possible. In fact, the principle of "prevention is better than cure" is also applicable to MASLD. If the development process of the damage can be blocked at an early stage, MASLD will be reversible. Fortunately, scientists have been focused on that excessive dietary intake may be a primary contributor to the early onset of MASLD. More interestingly, by adjusting dietary habits to focus on the intake of nutritional elements, rather than excessive or unhealthy foods, it may be possible to treat MASLD and potentially prevent its occurrence [6].

Here, we aim to reveal the close relationship between diet and MASLD through in-depth summary of articles related to dietary causing or dietary improving MASLD in the past five years. In addition to unraveling the pathological mechanism of MASLD caused by high-fat and high-sugar diet, based on the pathological mechanism, we will explore the painless and effective treatment of MASLD through dietary intake, and enrich the therapeutic methods of MASLD.

## Dietary models of MASLD

Admittedly, an unhealthy diet contributes to obesity, which is a significant and prevalent risk factor for MASLD. Over the past few decades, researchers have demonstrated that these dietary models of MASLD reflected many of the physiological, pathological, and biochemical characteristics of human MASLD [7]. Given that MASLD is a highly complex disease, we will delve into the key findings and disease processes of these most widely used and purely diet-driven models of MASLD, focusing on high-fat diet (HFD), choline-deficient high-fat diet (CD-HFD), choline-deficient, L-amino acid-defined, high-fat diet (CDAHFD), choline-deficient L-amino acid-defined diet (CDAAD), high-fat high-cholesterol diet (HFHCD), methionine-choline deficiency diet (MCD), high-fat methionine- and choline-deficient diet (HF-MCD), and western diet (WD), and discuss the predisposition of each diet to pathological manifestations, to provide some help for clinical treatment.

### HFD-induced MASLD models

**Fig. 1:**
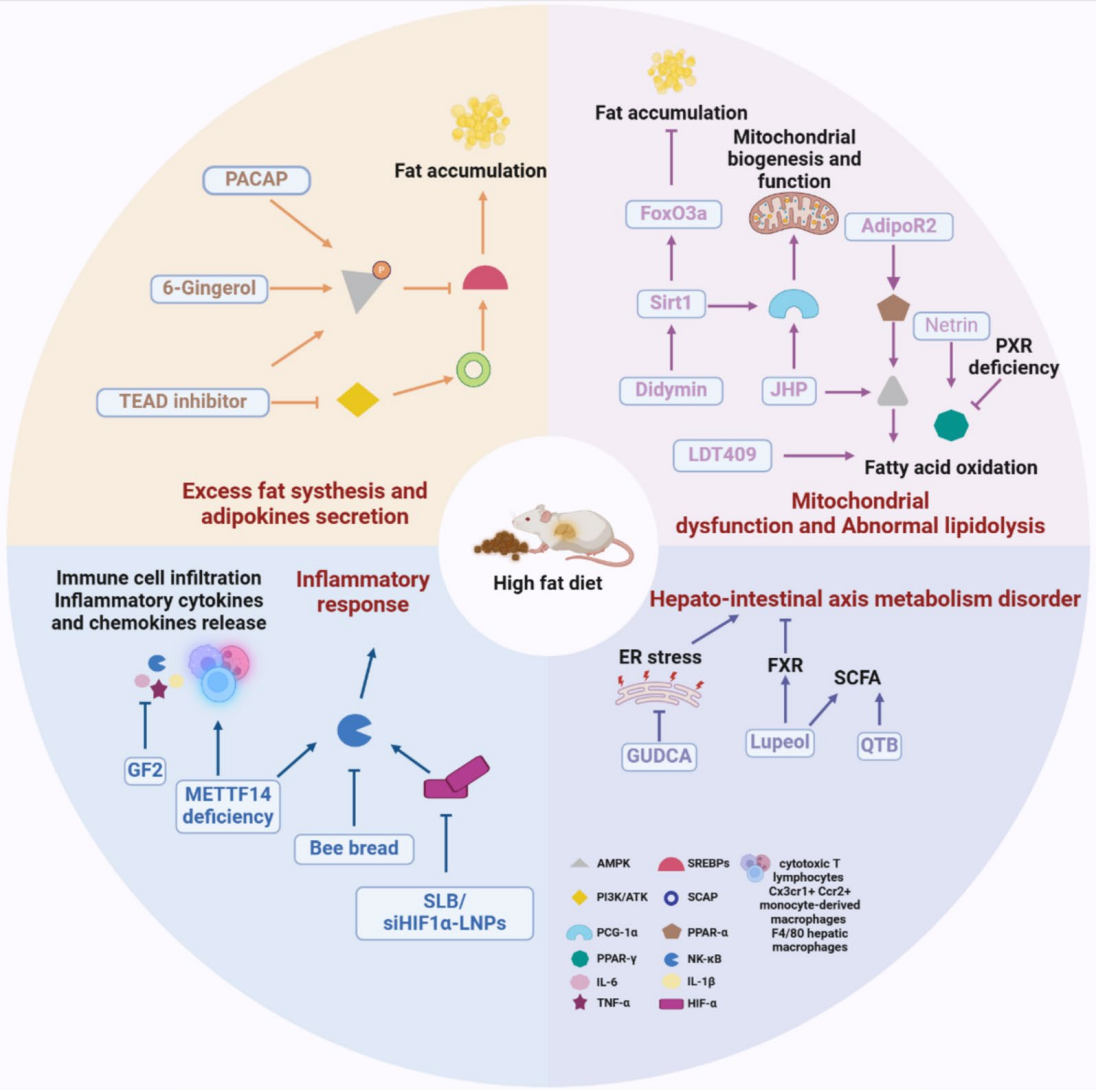
The pathological mechanisms of MASLD induced by a high-fat diet, including fat accumulation, mitochondrial dysfunction, inflammation, and hepato-intestinal axis disorders. Key pathways (AMPK, PPARs, SREBPs) are highlighted for their roles in regulating lipid metabolism, mitochondrial function, and inflammatory responses

In general, organisms are able to efficiently synthesize and break down lipids through lipid metabolism regulatory networks to maintain energy balance and substance metabolism in the body (Fig. 1).

#### How does sterol regulatory element-binding proteins (SREBPs, a key component of lipid synthesis) regulate the MASLD process?

Digestion, absorption and synthesis are the first steps in the process of fat metabolism, while lipid synthesis is dysfunctional after HFD stimulation. SREBPs, as vital regulator of lipid synthesis and uptake, are activated by SREBP cleavage-activating protein (SCAP) to exert transcriptional activity [8]. Du et al*.* revealed that combined exposure to PM2.5 and HFD significantly increased ROS levels to upregulate lipogenesis-related genes such as SREBP1, liver X receptor α (LXRα), SCD1, fatty acid synthase (FAS), and acetyl-CoA carboxylase 1 (ACC1) and the subsequent upregulate miR-155 expression caused the downregulation of peroxisome proliferator-activated receptor γ (PPARγ), ultimately promoting hepatic lipid metabolism disorders and steatosis [9]. The activation of the hepatic A1 adenosine receptor (A1AR) inhibited the maturation of SREBPs by reducing SCAP content and its anchoring in the Golgi apparatus, while prevented the phosphorylation and nuclear translocation of mature SREBPs, particularly SREBP1c and SREBP2 by decreasing adenylate cyclase activity to reduce cAMP levels and lower protein kinase A catalytic subunit (PKAc) activity, eventually, reducing lipid accumulation, and slowing progression of MASLD caused by HFD [10].

Attentively, AMP-activated protein kinase (AMPK) signaling pathway, like PKAc signaling pathway, regulates the shear activation of SREBP-1. In HFD-fed mice, the up-regulation of FAS apoptosis inhibitory molecule (FAIM) levels by pituitary adenylate cyclase-activating polypeptide (PACAP) activated downstream effector AMPK and insulin receptor β (IRβ) phosphorylation (a key regulatory element of metabolism) to cause the decreased expression of SREBP1/2, improving aberrant lipid metabolism and energy surpluses [11]. Subsequently, Xia et al*.* also confirmed the mechanism by which AMPK regulated lipid metabolism, AMPK phosphorylation was activated by 6-gingerol to suppress the nuclear translocation of SREBPs, thereby inhibiting hepatic steatosis to regulate lipid metabolism in HepG2 cells treated with palmitate (PA) [12]. Likewise, the phosphoinositide 3-kinase/protein kinase B (PI3K/ATK) pathway also can regulate lipid metabolism (de novo lipogenesis) by activating SCAP to facilitate SPEBP1/2 [8]. TEA domain family member 1 (TEAD1), a key transcription factor in the Hippo pathway, has recently been identified as a critical pathway for liver homeostasis and metabolism, was found to significantly upregulation of TEAD1 in MASLD patients. TEAD inhibitors (estrogen receptor, ER) significantly altered metabolic pathways, such as AMPK and PI3K/AKT signaling and reduced the expression of lipid metabolism related genes such as SREBP1 and 3-hydroxy-3-methylglutaryl-Coenzyme A reductase (HMGCR) (responsible for controlling cholesterol synthesis) by directly binding to their promoters to alleviate hepatic steatosis in HFD-induced MASLD male mice [13]. However, in adult offspring mice fed HFD, injected with nicotine can increase transforming growth factor (TGF)-β1 and PI3K/ATK signaling pathway activity to aggravate IR, while inhibit the expression of SREBP1c and PPARα to impair lipid metabolic pathways [14]. This suggests that nicotine-induced lipid metabolism disorders and HFD-induced lipid damage mechanisms are not identical, and still needs to be further explored.

At the same time, researchers also focused on another type of lipid sensor, constitutive androstane receptor (CAR). Although both SREBP and CAR belong to the nuclear receptor superfamily, SREBP is mainly involved in the metabolic regulation of cholesterol and fatty acids, while CAR is mainly responsible for the metabolism and detoxification of exogenous chemicals. CAR does not directly regulate lipid synthesis, but influences lipid homeostasis through interactions with the nuclear matrix protein matrin-3 [15). In HFD-induced MASLD mice model, matrin-3 deficiency led to a reduction in the expression of CAR in the liver, resulting in carboxylesterase 2a (Ces2a) decrease to facilitate lipid accumulation meanwhile I interleukin 1 receptor (IL-1R1), signal transducer and activator of transcription 3 (STAT3) phosphorylation increase to facilitate phase response [15]. Other studies further elucidated that STAT3 was overactivated by HFD in MASLD model to prevent membrane translocation of glucose transporter 4 (GLUT4) and cholesterol metabolism in hepatocytes by inhibiting VAV3 expression, thereby worsening overall glucolipid metabolic disturbances [16].

#### How does PPARs (a transcription factor of lipid decomposition) participate in the MASLD process?

Under normal circumstances, dietary and hepatic TG could be decomposed into glycerol and FAs, among which, FAs enter the mitochondria to undergo oxidation, generating a significant amount of acetyl-CoA and ultimately releasing energy. Nevertheless, in HFD-fed mice showed the obvious rise of liver weight, as well as the elevated hepatic lipid droplets, the accumulated TG, the deposited total cholesterol (TC) [17], which may be related to impaired mitochondrial function due to exogenous intake of excess lipids. Yang et al. pointed out that didymin enhanced the expression and activity of Sirtuin 1 (Sirt1), a critical nicotinamide adenine dinucleotide (NAD)-dependent deacetylase, resulting in several beneficial effects to alleviate MASLD. Concretely, Sirt1 promoted mitochondrial biogenesis and function by deacetylating peroxisome proliferator-activated receptor gamma coactivator 1-α (PGC-1α), enhanced lipophagy and reduced lipid accumulation through the deacetylation of forkhead box O3a (FoxO3a) in HFD-induced MASLD mice model [18]. According to another study, Jiuzhuan Huangjing Pills (JHP) extract normalized mitochondrial metabolism by restoring ATPase activity, and mitochondrial complex function, additionally, mitigated hepatic lipid metabolism and oxidative stress by upregulating the expression of key genes involved in fatty acid metabolism, such as PGC-1, acyl-CoA dehydrogenase long chain (ACADL) and PPAR-α in HFD-induced MASLD mice model [19].

Emphatically, PPARs participate in a variety of biological activities, are divided into three subtypes, α, β/δ and γ [20]. Choi et al. demonstrated that netrin-1 expressed macrophages could upregulate PPARγ expression by activating the unc-5 netrin receptor B (UNC5b) receptor to promoted autophagy through increasing LC3 conversion, p62 degradation, and autophagosome formation, suppressing hepatic steatosis in HFD-induced MASLD mice model [21]. In contrast, Sun et al. showed that the inhibition of hepatocyte autophagy, in a manner dependent on mTOR, contributed to the alleviation of MASLD to fibrosis through the upregulation resulting from the knockdown of tet methylcytosine dioxygenase 3 (TET3), which reduced ectonucleotide phosphodiesterase 1 (ENPP1) expression in HFD-induced MASLD mice model [22]. Excessive autophagy may lead to hepatocyte dysfunction, potentially explaining this differential outcome. With the deepening of research, some scientists have found that PPARs not only promote autophagy but also are tightly related to glucose homeostasis and lipid metabolism. HFD caused the disorder of PPARγ-related fatty acid synthesis and PPARα-dependent fatty acid oxidation in MASLD model, while, Taohe Chengqi Decoction (THCQ) enhanced branched chain amino acids (BCAA) catabolism to reduce phosphorylation of ATP citrate lyase (ACLY) to inhibit lipid synthesis, promoted lipid oxidation, and significantly alleviated MASLD [23]. Similarly, LDT409, a partial agonist of PPARα and PPARγ, promoted weight loss by reducing hepatic lipid accumulation and TG through upregulation of fatty acid oxidation genes fatty acid binding protein 1 (Fabp1), cluster of differentiation 36 (Cd36), cytochrome p450 family 4 subfamily A member 14 (CYP4A14), enhancing thermogenesis and fatty acid oxidation in brown adipose tissue uncoupling protein 1 (Ucp1), carnitine palmitoyltransferase 1 (CPT1), and inducing browning in Ucp1 in the HFD-induced MASLD mouse model [24].

In addition, the researchers also found that PPARγ can cooperate with AMPKα to regulate fatty acid oxidation [25]. Danthron upregulated adiponectin receptor 2 (AdipoR2) expression to, on the one hand, activate the AMPKα pathway and, on the other hand, enhance the nuclear translocation of the PPARα/retinoid X receptor (RXRα) heterodimer and bind with the AdipoR2 promoter, thereby promoting fatty acid oxidation, mitochondrial biogenesis, and energy expenditure in HFD-induced MASLD mice model [26]. Gebreyesus et al. also demonstrated that female mice with HFD-induced MASLD exhibited the upregulation of PPARγ and the downregulation of anti-obesity genes AMPKα, cytochrome P4502a5 (*Cyp2a50*), Aldo–keto reductase family 1 member B7 (*Akr1b7*), Acyl-CoA oxidase 1 (*Acox1*), but the deletion of pregnane X receptor (*Pxr*) as a regulator of glycolipid metabolism could suppress MASLD [27].

#### How does inflammation in HFD contribute to MASLD progression?

Excessive lipid accumulation in the liver also activates pro-inflammatory cells and the release of pro-inflammatory factors, such as tumor necrosis factor α (TNF-α) and nuclear factor-kappa B (NF-κB), resulting in more severe inflammation and lipid damage. NF-κB is a crucial regulator of the immune response, can be activated by oxidative stress and pro-inflammatory cytokines [28]. HFD-induced MASLD mice model showed increased cytotoxic T lymphocytes and pro-inflammatory markers (hypoxia-inducible factor-1 α (HIF-1α), TNF-α, NF-κβ) and lowered regulatory T cell (Treg) population and the anti-inflammatory marker interleukin (IL)-10 [29]. Bee bread reversed the increase of pro-inflammatory NF-κB in HFD-induced MASLD mice [30]. Furthermore, a hepatocyte-targeted nanoparticulate formulation (silibinin (SLB) /siHIF1α-LNPs) inhibited the HIF-1α/NF-κB pathway by silencing HIF-1α, while modulated immune responses to balance Tregs and cytotoxic T cells [29]. The latest evidence pointed out that oxidative low-density lipoprotein (oxLDL) activated the NF-κB signaling pathway in double negative T (DNT) cells through CD36 receptors, upregulated the expression of HIF-1 α and ACSL4, induced ferroptosis in DNT cells, thereby disrupting liver immune homeostasis and accelerating the progression of MASLD to fibrosis and cirrhosis [31]. Furthermore, METTL14 is known as a key m6A methyltransferase. According to another study, in HFD-induced MASLD mice model, the downregulation of methyltransferase-like 14 (METTL14) decreased glutaminase 2 (GLS2) protein levels by reducing N6-methyladenosine (m6A) modification of GLS2 mRNA, which exacerbated oxidative stress in the liver. Concurrently, this deficiency attracted Cx3cr1^+^Ccr2^+^ monocyte-derived macrophages that expressed myeloid differentiation primary response gene 88 (MyD88) to activate the NF-κB pathway, thereby amplifying inflammation and stimulating MASLD progression [32]. Ginsenoside F2 (GF2) also showed beneficial effects in inhibiting macrophages accumulation in HFD-induced MASLD mice model, inhibited LXR activity and decreased F4/80 hepatic macrophage infiltration and inflammatory cytokines (IL-1β, TNF and IL-6) expression, thereby exerting anti-steatosis and anti-inflammatory effects [33].

#### How does the hepato-intestinal axis participate in MASLD progression?

The occurrence of MASLD may be associated with the damage to the intestinal barrier and disruption of gut-liver axis. Intestinal flora can synthesize bile acid (BA) and short-chain fatty acid (SCFA) to regulate MASLD. Glycoursodeoxycholic acid (GUDCA) is BA metabolites, can significantly reduce ER stress markers such as C/EBP Homologous Protein (CHOP), phosphorylated inositol-requiring enzyme 1 (p-IRE1), phosphorylated p38 MAPK (p-p38), and phosphorylated c-Jun N-terminal kinase (p-JNK). Additionally, GUDCA partially decreased phosphorylated eukaryotic initiation factor 2 α (p-eIF2α) expression, and also alleviated metabolic disorders by restoring sarcoplasmic reticulum calcium ATPase 2 (SERCA2) expression to maintain intracellular calcium homeostasis in HFD-induced MASLD mice [34].

Farnesoid X receptor (FXR) is a BA binding transcription factor that is activated to control lipid, glucose, and energy metabolism. In HFD-induced MASLD mice model, lupeol activated FXR to downregulate SREBP-1c and FAS, which reduced hepatic lipid accumulation and promoted lipid clearance. Additionally, lupeol enhanced FXR-mediated expression of bile salt export pump (BSEP) and reduces CYP7A1, facilitating BA excretion and preventing cholestasis. Furthermore, it modulated FXR to decrease IL-6 and increase IL-10, which in turn reduced liver inflammation and fibrosis and regulated apoptotic pathways to promote the removal of damaged hepatocytes. Concurrently, lupeol improved gut microbiota composition, increased SCFAs production, and enhanced intestinal barrier integrity through FXR activation [35]. Similarly, theabrownin extracted from Qingzhuan Tea (QTB) increased the relative abundance of beneficial intestinal microbiota such as *Bacteroides, Blautia, and Bachnoclostridium*, while decreased the abundance of intestinal microbiota linked to obesity, like *Colidextribacter, Faecalibaculum*, and *Lactobacillus*. QTB significantly increased the levels of the SCFAs and production such as acetate and propionaten in HFD-induced MASLD mice model. Additionally, QTB treatment resulted in significant changes in hepatic metabolic signals by increasing the expression of genes like ATGL, PPARα, the short-chain fatty acid receptors (FFAR2 and FFAR3), which contributed in improving liver metabolism and preventing MASLD [36]. Studies have further revealed the mechanism by which HFD-induced dysregulation of the gut-liver axis affects BA metabolism and immune responses, thereby accelerating the progression of MASLD. HFD disrupted the gut microbiota by increasing the abundance of *Clostridium* and *Bilophila*, which in turn elevated secondary BAs (DCA, LCA (lithocholic acid) and dysregulated the FXR-FGF19 axis, leading to excess BA synthesis, lipid accumulation, and inflammation. Moreover, DCA-induced Takeda G-protein-coupled receptor 5 activation enhanced Kupffer cell activation to increase TNF-α and IL-6 levels and aggravate liver damage [37]. Also, HFD was reported to weaken intestinal barriers by reducing MUC2, ZO-1 and occludin, increasing gut permeability and LPS translocation, which then activated TLR4/NF-κB signaling and promoted M1 macrophage polarization. Additionally, HFD altered BA-immune interactions, leading to Kupffer cell and CD8⁺ T cell dysregulation, hepatic stellate cell (HSC) activation and fibrosis through FXR and TGR5 signalings [38]. These indicated that BAs played a key regulatory role in the development of MASLD by effecting immune microenvironment.

### CD-HFD/CDA-HFD/CDAAD-induced MASLD models

Based on HFD diet, researchers have developed a range of new experimental animal models to further explore the mechanisms and therapeutic agents of MASLD disease. MASLD model in mice fed CD-HFD had a pathological manifestation of obesity, collagen deposition, IR, hepatic steatosis, and aberrant lipid metabolism. Elafibranor, a dual agonist of PPAR α and δ, increased hepatic PPARα ligand and upregulated S100A4 protein levels, thereby inducing the epithelial-mesenchymal transition program, additionally, decreased the levels of α-smooth muscle actin (α-SMA) and collagen type I α1 (COL1A1) to alleviate the progression of MASH like fibrosis in CD-HFD mice [39].

Another model, while similar to CD-HFD, known as CDA-HFD, induces the formation of MASLD through a combination of choline and specific amino acid deficiencies alongside a high-fat diet. This model has been shown to induce NASH and liver fibrosis in animals over an extended period. These male mice fed with CDA-HFD also showed the increased mRNA expression of Col1a1 and α-SMA, indexes of collagen accumulation and fibrosis in liver tissue. However, administering compound 11c, a peripheral antagonist of 5-hydroxytryptamine_2A_ (5HT_2A_), can significantly change the above situation [40]. Research has shown that the administration of fibroblast growth factor 21 (FGF21) can inhibit protein expressions of collagen I and α-SMA to alleviate liver fibrosis [41]. Nevertheless, Rocío et al*.* found hepatic and circulating FGF21 expression increased in mice fed CDA-HFD, as a matter of fact, the A-allele from the re838133 variant located in the FGF21 due to diet is more susceptible to MASLD and progress to fibrosis [42]. In addition to the pathological manifestations of fibrosis, CDA-HFD also caused macrophage pyroptosis to further expand the inflammatory response. Triggering receptor expressed on myeloid cells 2 (TREM2) was expressed by MASH-associated macrophages, an indicator of inflammation and fibrosis progression. When TREM2 were overexpressed, the macrophage pyroptosis can be inhibit through PI3K/AKT pathway and the macrophage can be reprogrammed by up-regulating transforming growth factor (TGF)-β1 (a main inducer of macrophage M2 polarization) to anti-inflammatory phenotype, thereby treating MASLD [43].

In the CDAAD-induced MASLD mice model, which utilizes amino acids to replace all proteins while avoiding the introduction of choline found in proteins, histidine-rich glycoprotein (HRG) was observed to be significantly upregulated. This upregulation resulted inthe downregulation of IL-10, and TREM2 as well as an increase in pro-inflammatory cytokines IL-1β, IL-6, IL-12 and angiogenic factors such as VEGF-A. These changes facilitated the recruitment and activation of macrophages and myofibroblast-like cells in the liver, leading to increased collagen deposition and the progression of liver fibrosis. However, the deletion of HRG retarded the progression of MASLD-related liver carcinogenesis [44]. In summary, our findings indicate that the diet structure of CD-HFD/CDA-HFD/CDAAD is effective for constructing a pathological model of MASH development into fibrosis.

### HFHCD-/HF-MCD-induced MASLD models

In order to better simulate human MASLD, researchers induced MASLD animal models based on a high-fat diet supplemented with high cholesterol or methionine-and choline-deficient to study the complex pathological mechanism of MASLD. Similar to the HFD model, HFHC-induced mice showed abnormal AMPK/SREBP lipid metabolic pathway, interestingly, Si-Ni-San (SNS) can activate AMPK to inhibit fatty acid synthase (FASN) expression to reduce lipid accumulation, while these effects being counteracted by AMPK inhibition [45]. In addition to lipid metabolism disorders, studies have suggested that MASH may drive systemic inflammatory responses, leading to neuroinflammation and thus cognitive dysfunction. The HFHCD caused an increase in monocyte chemoattractant protein 1 (MCP-1), IL-6 and IL-17 in mice, causing macrophages released MIP-1α to drive systemic inflammation. Also, this simultaneous increased in the cytokines C-X-C motif chemokine ligand 1 (CXCL1) and promoted neurodegeneration caused by microglia activation [46]. Therefore, timely intervention in the inflammatory response of MASH is particularly vital. Cui et al. research has shown that the gas transport agent CO released by tyrene maleic acid copolymer encapsulating CO-releasing molecule (SMA/CORM2) led to the inhibition of classical activated macrophages (M1) polarization in macrophages and the activation of NOD-like receptor protein 3 (NLRP3) inflammasome, modulated MASLD induced by HF-MCD though the significant inhibition of HIF-1α [47].

### WD-induced MASLD models

WD, characterized by high levels of animal and trans fats, sugar and fructose, and low levels of vegetables, is one of the most common diets to induce the MASLD model. Apolipoprotein E (ApoE) plays a crucial role in lipid metabolism and the regulation of lipid homeostasis by binding low density lipoprotein receptor (LDL-R). ApoE^−/−^ mice fed WD showed the increased expression of CD36 and LDL-R genes, which promoted the uptake of long-chain fatty acids, while concurrently showing decreased expression of Adipor2 in liver, thereby impairing lipid metabolism. However, lupin protein hydrolysates (LPHs) can reverse the above situation and improve MASLD by reducing levels of hepatic cholesterol [48]. Under the influence of cholesterol and chemokines produced by liver cells, neutrophils release hepatocyte growth factor (HGF), thereby accelerating liver repair and regeneration [49]. Burbano et al. proven that in primary hepatocytes of MASLD mice fed with WD, HGF improved mesenchymal-epithelial transition (MET) phosphorylation, which in turn accelerates liver cell proliferation, so basal MET phosphorylation was used to detect liver health and predict the risk of liver failure [50].

### MCD-induced MASLD models

In another common animal model of MASLD, MCD model, essential nutrients required for phospholipid synthesis are removed, leading to significant fat accumulation in the liver and the development of fatty liver disease. Yan et al*.* found that Yinhuang granule (the traditional Chinese medicine) effectively reduced the fat accumulation, inflammation, oxidative stress and alleviated MASLD in MCD-fed mice through the activation of nuclear factor E2-related factor 2 (Nrf2), which is a regulator of cellular oxidative stress in MCD -induced MASLD model [51]. Attentively, oxidative stress could facilitate Nrf2 to recruit the SRBEP-1 promoter to increase SREBP-1-mediated lipogenesis [52]. In both the HFD and MCD-induced MASLD mice models, the expression of hepatic lipogenesis genes SREBP1 and the glucose transporter (GLUT9) expression were significantly downregulated for attenuating MASLD symptoms after knockouting GLUT9 in liver [53]. Furthermore, Luo et al*.* observed that another enzyme involved in glycolysis, aldehyde dehydrogenase 2 (ALDH2), which also played an important role in oxidative stress and apoptosis, especially in MASLD patients and MCD-induced MASLD mice. ALDH2 level was remarkable reduction while ALDH2^−/−^ in MCD-fed mice generated the decrease expression of oxysterol 7-α hydroxylase (Cyp7b1) and the related metabolic pathway FXR/SHP to impair BA metabolism [54]. Of particular concern is the fact that iron-dependent lipid peroxidation also causes programmed cell death. In general, glutathione peroxidase 4 (GPX4), as the negative controller of ferroptosis, comprises both cGPX4 and iGPX4. Tong et al*.* showed that, in MCD-fed MASLD mice, the knock-in of cGPX4 relieved steatohepatitis and liver injury, whereas the knock-in of iGPX4 produced the opposite result. Interestingly, the iGPX4 subtype directly interacted with cGPX4, contributing to the formation of a high-molecular enzyme complex that inactivated GPX4, thereby facilitating ferroptosis in MASLD mice [55].

MASLD is a dynamic and multifactorial disease characterized by the gradual emergence of metabolic complications such as IR, alongside its pathological features, which cause irreversible damage together. Consequently, it is essential to identify more effective and appropriate treatment methods for individuals, guided by the therapeutic targets established in existing studies.

## Dietary therapy of MASLD

Emerging evidence indicates that the dietary patterns of patients with MASLD, characterized by a high intake of added sugars, sugary beverages, processed foods, and pastries without alcohol are associated with pro-inflammatory responses and an increased risk of severe hepatic steatosis [56]. Latino individuals carrying the homozygous guanine (GG) genotype variant of the PNPLA3 gene (coding for triacylglycerol lipase) were more susceptible to MASLD, and the risk of developing cirrhosis in MASLD patients with GG carriers was positively associated with high sugar intake such as total sugar, fructose, sucrose, and glucose [57]. Coincidentally, Rispo et al*.* also proved that MASLD patients carrying the PNPLA3 gene variant exhibited more severe metabolic abnormalities, such as elevated liver enzymes, higher LDL cholesterol, and increased IR, compared to non-MASLD patients [58]. Nevertheless, an 8-week diet limiting free sugars in adolescent boys resulted in a 10% reduction in DNL and a 7% reduction in liver fat, and improved metabolic parameters such as fasting insulin and TG [59].

### New strategies for dietary managing MASLD, the scientific mystery of the ketogenic diet (KD)

In fact, in order to effectively combat the further damage caused by saccharides to MASLD patients, KD, as a new low-carb diet, has been developed. KD is composed of 3–5% carbohydrates, 20–27% proteins and 70–75% of lipids, can induce nutritional ketosis that breaks down fat to produce energy as well as decrease blood sugar levels through reduced carbohydrate intake, improving insulin sensitivity and achieving weight loss goals in overweight or obese patients. In HFD-induced MASLD model, KD induced interleukin 6 (IL-6) production and thus activated c-Jun N-terminal kinase (JNK), which in turn inhibited insulin signal redirection, thereby preventing MASLD [60]. To improve MASLD more effectively by reducing CMRF (BMI, serum glucose, blood pressure, plasma triglycerides, plasma HDL-cholesterol) and MASH, a very low-calorie ketogenic diet (VLCKD), advised to consume 2 L of water and 800 kcal of calories per day, has emerged. The patient with obesity or overweight treated with VLCKD, showed the reduction of waist circumference, fat mass, blood pressure, hepatic steatosis and fibrosis. In addition, further research found that VLCDK reduced inflammatory expression mainly by reducing the counts of white blood cell (WBC) and platelet (PLT) [61]. As a matter of fact, VLCDK therapy can substantially reduce the symptoms of liver steatosis and CMRF in overweight and obese patients, especially the subject with a high level of steatosis and fibrosis before diet had more obviously decrease [62]. Observingly, the data on overweight or obese patients indicated that men exhibited higher levels than women. This disparity persisted even after the administration of VLCDK treatment, which may be attributed to differences in hormonal and metabolic factors between the sexes [63].

### The positive influences of the healthy dietary pattern and diet scoring system on MASLD

Furthermore, Zhang et al*.* analyzed the dietary patterns and health outcomes of 421 adults, primarily of Mexican and Central American origin, with an average age of 45 years and a high prevalence of obesity (BMI 35.0 kg/m^2^), type 2 diabetes (66.8%), and hypertension (41.6%). The fast-foods/meats pattern was associated with a 2.47-fold increased risk of severe liver steatosis compared to the plant-foods/prudent pattern, highlighting the significant impact of dietary habits on liver health [64]. The Mediterranean diet (MD) is widely recognized for its emphasis on plant-based foods, complemented by moderate quantities of olive oil, healthy dairy products, and fish, which collectively offer anti-inflammatory and antioxidant benefits for patients. The patients followed two plant-foods/prudent dietary strategies, the American Heart Association (AHA diet, 55% carbohydrates, 15% protein, 30% of lipids) and the MD diet (40–45% carbohydrates, 25% proteins, 30–35% of lipids). The results showed that both of them could inhibit the inflammatory markers, among markers leptin, adiponectin, M30 and LECT2 [65]. Other study demonstrated that MD significantly reduced TC and liver stiffness in MASLD patients [66]. Additionally, it significantly mitigated inflammation induced by oxidative damage significantly by reducing plasma MDA levels and lowering the release of pro-inflammatory cytokines (CRP, IL-1ra, and MCP-1), furthermore, attenuated TLR4-mediated hepatic inflammation by reducing plasma LPS and zonulin levels in MASLD patient [67]. Attentively, adhering MD treatment well had a lower MASLD risk in patients with the minor allele of rs780094 variant located in the glucokinase regulatory protein (GCKR) gene, which can oppose effects on glucose and triglyceride metabolism [68].

As research advances, scholars are increasingly convinced of the impact of dietary factors on disease progression. Consequently, a dietary scoring system has been established to quantitatively assess the relationship between dietary factors and disease risk, thereby facilitating the development of effective health interventions for patients. The Alternative Mediterranean Diet (AMED) score was evaluated among veterans of different races and nationalities and the results showed that AMED score was inversely associated with MASLD, which was related to BMI and total energy intake [69]. Huang et al*.* found that Alternate Healthy Eating Index (AHEI), Dietary Approaches to Stop Hypertension (DASH) score and AMED score were negatively correlated with the risk of MASLD. For example, the participants with the highest tertile of AHEI reduced 30% risk of MALD, and once AHEI, DASH and AMED scores increased 20%, the risk of MASLD decreased by 43%, 52% and 52%. Among the three healthy eating patterns, vegetables and whole grains are the main parts to improve MASLD [70]. Ulteriorly, Jain et al*.* study found that a higher HEI was associated with lower body weight, higher serum HDL cholesterol, and lower serum triglycerides, suggesting that a healthier diet roughly included fruits, whole grains, vegetables and lean meats, may help mitigate metabolic risk factors in children with MASLD [71].

### New strategies of dietary therapy for MASLD, intermittent fasting (IF)

To enhance the treatment of MASLD, researchers are actively investigating the impact of various dietary therapies on the progression of the disease. The 5:2 diet, a form of IF, involves two days of strict calorie restriction per week, followed by five days of normal eating. Michelle Y. Lewis et al*.* recruited participants from different races and ethnic backgrounds, randomly arranged diets, including intermittent energy restriction (5:2 Diet) and energy restriction plus the Mediterranean dietary (intermittent/daily energy restriction, IER/DER + MED), and physical activity intervention to explore the diet pattern and strategies for reducing visceral obesity. IER + MED can promote the reduction of visceral adipose tissue (VAT) to alleviate the obesity epidemic and its metabolic sequelae [72]. In the CD-HFD-induced MASLD mouse model, the mechanism by which dietary restriction (5:2 diet) to treat disease by activating PPARα was validated. The upregulated PPARα involved in mitochondrial biogenesis and ketogenesis, specifically through the action of phosphoenolpyruvate carboxykinase 1 (PCK1), which reduced hepatic triglycerides and overall liver steatosis. Additionally, PPARα effectively alleviated liver inflammation and inhibited liver fibrosis by promoting fatty acid oxidation (CYP4A10, CYP4A14, ACOT2(acyl-CoA thioesterase 2) and CROT (carnitine O-Octanoyltransferase) and suppressing lipogenesis, which was crucial in slowing the progression of hepatocellular carcinoma (HCC) [73]. In addition, time-restricted feeding (TRF) has attracted attention because of its simplicity and effectiveness in maintaining circadian rhythms, reducing body weight and improving metabolism. TRF restored the diurnal oscillations of beneficial microbial genera that were disrupted by WD, such as *Lactobacillus*, *Romboutsia*, and *Dubosiella*. The restored microbial rhythmicity led to a cyclical production of these beneficial metabolites, like indole derivatives, which in turn reduced hepatic inflammation, steatosis, and liver fibrosis [74]. In MASLD patients, according to a recent study, IF, particularly Ramadan fasting (nocturnal feeding) and Alternate-Day Modified Fasting (ADMF), led to significant reductions in body weight, BMI, and, as well as improvements in metabolic markers such as HDL-C, blood pressure, IR and lipid profiles in patients with MASLD [75].

### New strategies of dietary therapy for MASLD, dietary supplement (nutritional agents)

Despite the numerous advantages associated with restricted diets, patients may encounter challenges, such as difficulty adhering to these diets or experiencing deficiencies in certain nutrients. Additionally, dietary supplements for the adjuvant treatment of MASLD offer a novel perspective.

Coffee, as a dietary supplement, contains a variety of nutrients, including B vitamins, antioxidants, proteins, fats, and dietary fiber. In patients with MASLD, consuming two or more cups of coffee per day significantly reduces the risk of developing primary liver fibrosis, particularly among those who consume between two and three cups daily [76]. Mechanically, caffeine can protect primary rat hepatocytes from PA toxicity by inhibiting A1AR activity, and increasing cyclic adenosine monophosphate (cAMP) levels through the activation of PKA and extracellular signal-regulated kinase 1/2 (ERK 1/2) signaling pathways [77]. As mentioned earlier, A1AR ultimately improves lipid accumulation by preventing phosphorylation and nuclear translocation of mature SREBPs. The research demonstrated that the combination of vitamin D3 (VD3) and omega-3 oils therapy also showed better therapeutic effectiveness against MASLD, enhanced glucose and lipid metabolism and antioxidant and anti-inflammatory liver by improving obesity symptom and metabolic characteristics, reducing hepatic triglyceride esters, inhibiting PPARs, SREBP1, increasing insulin-induced gene 1 (INSIG1), adiponectin, AdipoR1, leptin, leptin receptor (LEPR) in HFFD model [78]. In addition, intervention with VD3 and VC can similarly reverse dysbacteriosis by regulating the composition of the gut microbiota, such as *Allobaculum, Faecalibaculum, and Lachnospiraceae*_*UCG-006* in HFD-fed MASLD mice, likewise, enhance BA metabolism in the gut-liver axis by increasing the expression of BA synthesis-related genes CYP7A1 and BA transport-related genes FXR and BSEP in the liver while decreasing the expression of apical sodium-dependent bile acid transporter (ASBT) in the intestine [79]. The evidence suggested that treatment with serum 25-hydroxy VD alone can also reduce the risk rate of MASLD patients [80]. Concretely, VD supplementation in MASLD patients resulted in significant improvements in metabolic profiles and inflammation by upregulating levels of lipid profiles (HDL-C) and downregulating levels of liver enzymes (ALT and AST), LDL-C, and reducing pro-inflammatory microRNAs (MiR-21 and MiR-122) [81].

### The beneficial effect of dietary prebiotics and probiotics on MASLD treatment through hepatoenteric axis regulation

Generally, the non-free sugar, fiber and starch in whole grains may have a preventive effect on MASLD [82]. Hence, the evidence suggested short-term amylase/trypsin inhibitors (ATI)-free diet (refraining from high-gluten foods) significantly reduced liver fat content, improved IR on patients with MASLD [83]. Scientists deem that the benefits of this diet may be due to prebiotics, which are generally carbohydrates that selectively promote the metabolism and proliferation of beneficial bacteria in the body, thereby improving the organic matter of host health. Posteriorly, dietary fiber can increase the level of SCFAs (acetate, proprionate, butyrate) in MASLD patients, which are produced by specific bacteria such as *Faecalibacterium prausnitzi*i, and increasing beneficial bacteria, such as *Akkermansia muciniphila* and *F*.* prausnitzii* [84]. Other studies have also pointed out that in western standard diet (WSD)-fed mice, dietary oat beta-glucan (soluble dietary fiber) increased the abundance of beneficial bacterial populations, such as *Lachnospiraceae, Ruminococcaceae*, and *Lactobacillu* to product SCFAs, giving rise to restore intestinal homeostasis and led to reduced translocation of pathogen-associated molecular patterns, subsequently decreasing hepatic inflammation and steatotic liver disease [85]. Peculiarly, Rosas-Campos et al*.* confirmed high fat dietary supplement with *Opuntia ficus indica, Theobroma cacao (cocoa) Acheta domesticus* and *Akkermansia muciniphila* also has prebiotic effect by increasing the production of SCFAs to activate G- coupled receptor (GPRs) in MASLD mice and obese or overweight patients meanwhile reduced oxidative stress by up-regulating the expression of oxidoreductase and increasing the nuclear translocation of Nrf2, as well as improved IR and lipid metabolism by regulating the expression of various miRNA (including miRNA-34a, miRNA-102, and miRNA-33) as the diet contains polyphenols [86]. Notably, *Akkermansia* also promote L-aspartic acid transport from the gut to the liver by upregulating L-aspartate transporter (*Slc1a1 or Slc1a2*) expression, thus activating the liver kinase B1 (LKB1)-AMPK axis to inhibit lipid oxidation in HFHCD-induced MASLD mice model [87]. Another amino acid, lysine, also appears to be involved in lipid metabolism through epigenetic modification. In HFHCD mice, 3-HPAA (*Phocaeicola vulgatus* metabolite) inhibited histone acetylation, specifically lowering histone 3 lysine 27 acetylation (H3K27 ac) to suppress the expression of lipid metabolism-related genes like squalene epoxidase Squalene Epoxidase (SQLE), reducing liver fat accumulation in combating MASLD progression [88].

**Fig. 2:**
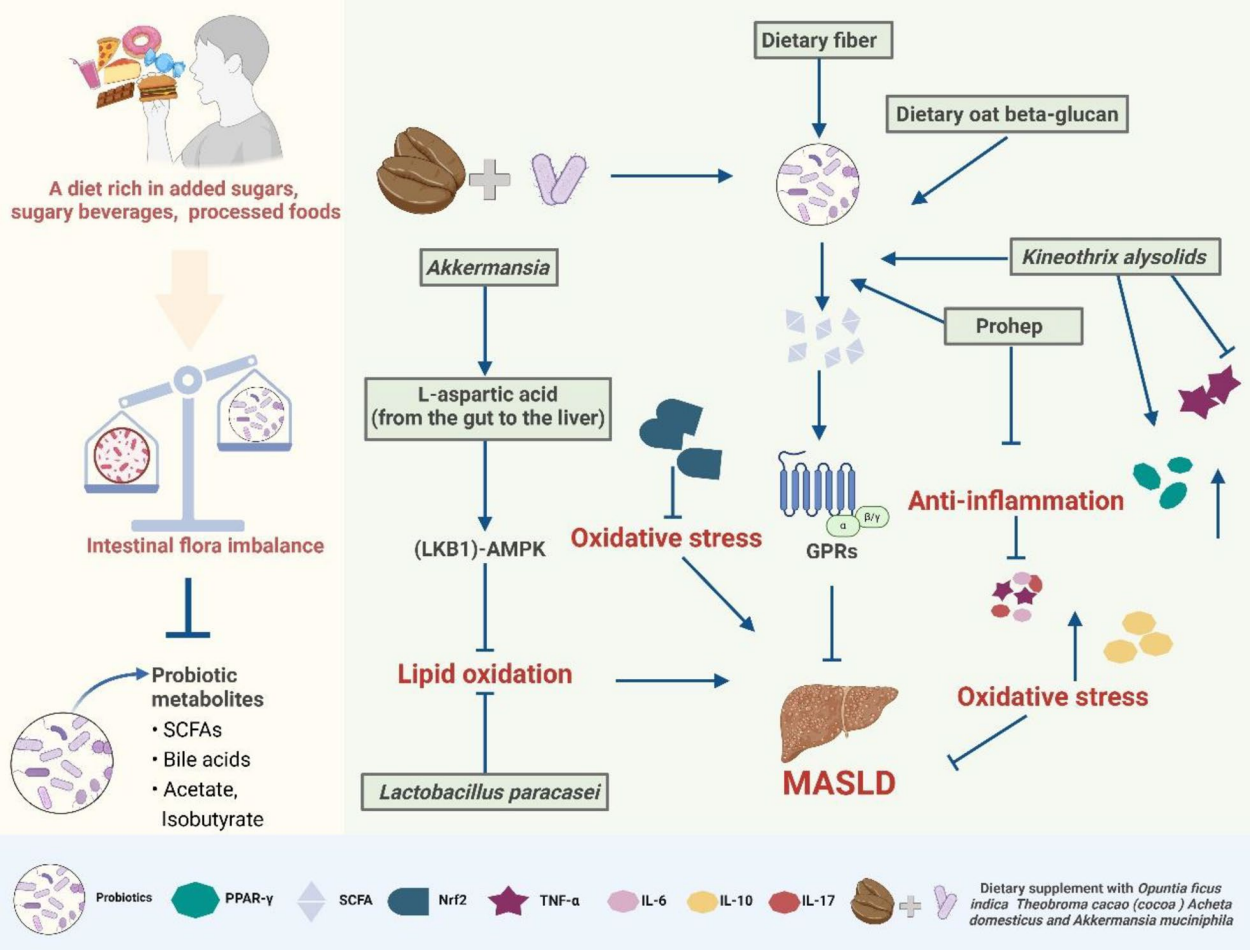
How dietary intake probiotics influences MASLD. A high-sugar, processed diet disrupts gut balance, promoting oxidative stress and MASLD. In contrast, dietary fiber and supplements (*Akkermansia*, *Prohep*, oat beta-glucan) restore gut balance, reduce inflammation, enhance fatty acid oxidation, and alleviate MASLD progression

Due to the interaction between the hepato-intestinal axis, an increasing number of researchers have begun to focus on the regulatory effects of probiotics on MASLD (Fig. 2). Larsen et al*.* demonstrated that another probiotic strains *Lactobacillus paracasei* was more effective in preventing weight gain, fat accumulation, and hepatic lipid accumulation in HFD-fed mice, whereas *L*.* paracasei* showed a more pronounced effect in fast food-mimicking diet (FFMD)-fed mice, particularly in improving glucose tolerance and reducing hepatic steatosis [89]. Besides, *Kineothrix alysoides* mitigated liver dysfunction by restoring gut microbial balance and reducing the abundance of harmful bacteria in mice subjected to a high-fat and high-fructose (HFHF) diet. Fructose serves as a significant factor in the progression of MASLD by upregulating malic enzyme 1 and acyl-CoA synthetase long-chain family member 1, which promoted hepatic de novo lipogenesis (DNL) and triglyceride accumulation, while enhancing Acyl-CoA synthetase short-chain family member 2-mediated acetate-to-acetyl-CoA conversion to amplify lipogenesis [90]. Concurrently, fructose disrupted gut microbiota composition, leading to an overgrowth of *Akkermansia* and a reduction in fungal diversity, which impaired intestinal barrier function and increased permeability. Furthermore, fructose facilitated LPS translocation that activated TLR4-dependent inflammatory pathways, ultimately elevating IL-6, TNF-α, and IL-17A levels [91].

Furthermore, it increased the presence of beneficial bacteria associated with improved liver health, such as *Acetatifactor, Roseburia,* and *Oscillibacter*. Additionally, *K*.* alysoides* upregulated the expression of lipogenesis-related genes like PPAR-γ and CD36 in the liver and reduced proinflammatory cytokine TNF-α expression in the colon, thereby exerting anti-inflammatory effects that contribute to mitigate liver dysfunction [92]. Similarly, the proprietary probiotic mixture (Prohep) improved hepatic lipid metabolism by downregulating key cholesterol metabolism genes and fatty acid metabolism genes, reducing the secretion of pro-inflammatory cytokines (IL-17, TNF-α and IL-6), and increasing the secretion of the anti-inflammatory cytokine IL-10, as well as led to a significant shift in the gut microbiota structure and increased the levels of SCFAs, specifically acetate and isobutyrate, which are beneficial for gut health in HFD diet-induced MASLD mice model [93]. Existing studies consistently indicate that both probiotics and prebiotics may play a significant role in the clinical treatment of digestive system diseases, serving as important dietary regulators, which presents promising clinical application prospects as well as opportunities for enterprise transformation and development.

## Discussion

Recently, MASLD has been redefined as a systemic disease that extends beyond the liver. The symptoms associated with each model, as documented in the investigated literatures, are summarized in Table 1. Based on this table, we can conclude that HFD (40–60% fat, 20–40% carbohydrates and 10–20% protein)-based models predominantly drive hyperlipidemia, hepatic steatosis and insulin resistance, accompanied by mild-to-moderate inflammation and oxidative stress within 12–20 weeks, closely mirroring early-stage human MASLD with CMRF such as hypertriglyceridemia, hyperglycemia, and hypertension. However, the severity of inflammation induced by HFD is proportional to the modeling time. Notably, all model animals based on a HFD are associated with obesity and IR. Lipid metabolism-related genes (PPARs, PI3K/AKT and AMPK/SREBPs signaling pathway, as well as their upstream and downstream regulatory targets) are the primary therapeutic focuses. It is important to note that TC in HFHC/HF-MCD can be deposited in hepatocytes and liver macrophages, exacerbating steatosis, oxidative stress damage, and immune-inflammatory response. HFHCD demonstrated the effect of cholesterol on modeling, inducing more severe inflammation associated with signaling pathways such as TLR4, TNF-α and IL-1β. On the other hand, choline-deficient models induced severe hepatic inflammation, oxidative stress and accelerated fibrosis with great rapidity via the up-regulation of TGF-β1/α-SMA and collagen deposition, without fully recapitulating obesity-driven insulin resistance. In MCD (10–15% fat (lard or soybean oil) and 40–50% carbohydrates)-induced MASLD model within 4–8 weeks, the levels of several fibrosis-related targets, *a-Sma*, *TGF-b1*, *Col1a1* and *Col3a1,* were significantly increased. The CDA-HFD and CDAAD are primarily MASLD models, induced by lipid accumulation and metabolic disorders, emphasizing choline deficiency and the potential to induce severe liver fibrosis in a short time (4–6 weeks) [94]. Besides, the choline-deficient high-cholesterol diet (CDHCD) model (1–2% cholesterol) induced hepatic cholesterol overload and steatosis within 4–12 weeks, yet its metabolic relevance was limited by paradoxical weight loss and suppressed PPARα-mediated lipid oxidation. In contrast, CDHFHCD (choline deficient high fat and high cholesterol) model (45% fat + 1% cholesterol) accelerated fibrogenesis in low-density lipoprotein receptor KO mice, achieving fibrosis by week 8 through dual mechanisms of hepatocyte apoptosis (caspase-3 activity increasing) and Kupffer cell activation [94]. Although both models aimed to simulate human MASLD, CDHCD did not replicate obesity-associated insulin resistance, as indicated by the unchanged HOMA-IR, whereas CDHFHCD model disrupted energy homeostasis via impairing thermogenesis and attenuating adipokine signaling, which was distinct from the metabolic processes observed in human MASLD. The differential progression of fibrosis in these models further underscores their distinct pathogenic mechanisms. WD (40–45% fat, 22% fructose and 1–2% cholesterol) is more closely to the models of obesity and metabolic syndrome, mainly inducing the disorders of mild liver function within 12–24 weeks. Given that WD is characterized by high levels of fat, sugar and cholesterol, it is also more likely to contribute to dyslipidemia and atherosclerosis. By identifying the disease symptoms and pathological tendencies of MASLD caused by different diets, researchers can select specific modeling methods in future studies, which will enhance the understanding of the occurrence and progression of MASLD and aid in the development of therapeutic strategies.

**Table 1:** Pathological features and key molecular targets of dietary MASLD models

Disease diet	Model	Variety	Gender	Age (week-old)	Diet in the article	Hepatic steatosis	Insulin resistance	Oxidative stress	Inflammation	Hepatic fibrosis	CMRF	Involvement cells					References
											Hypertriglyceridemia (Plasma triglycerides)	Hyperglycemia (Serum glucose)	Low high-density lipoprotein cholesterol (Plasma HDL-cholesterol)	Hypertension (Blood pressure)	Obesity (body weight)		
HFD	mice	C57BL/6J	Male	4 weeks	MCD, HFD	GLUT9/SLC2A9	GLUT9	–	–	–	↑	↑	–	–	–	Hepatocytes	[53]
	mice	C57BL/6	Male, female	8–10 weeks	HFD	PPARγ, Fsp27	–	Gstm3, Sod2, AMPK singaling pathway	Opn, Col1a1, Mmp2, Trem-1 singaling	Opn, Col1a1, Mmp2	↑	↑	–	–	↑	Hepatocytes, HSC, T Cells	[27]
	mice	C57BL/6J	Male	4 weeks	HFD	SREBP1, SREBP2, SCD1, FAS, HMGCR, AMPK-Irβ axis	IRβ, AKT	AMPK-mediated ROS suppression	FAIM-AMPK-Irβ axis	–	↑	↑	↓	–	↑	Hepatocytes	[11]
	mice, rat	C57BL/6J	Male	5–8 weeks (mice), 12–14 weeks (rat)	HFD	Adenosine Receptors (A1, A2A, A2B)	–	AMPK, ROS	PKA, Adenosine Receptor A1, A2A	PKA, cAMP-PKA singaling pathway	↑	–	–	–	–	Hepatocytes, HSCs	[77]
	mice	C57BL/6J	Male	10–12 weeks	CD-HFD	PPARβ/δ, PPARα	PPARβ/δ, FGF21	–	PPARβ/δ	PPARβ/δ, NF-κB, S100A4, EMT, TGF-β, α-SMA, COL1A1	–	↑	–	–	↑	Hepatocytes	[39]
	mice	C57BL/6J	Male, female	5 weeks	HFD	TEAD1	AMPK signaling pathway	Mitochondrial respiration	ERK signaling pathway	TGF-β signaling pathway	–	↑	–	–	↑	Hepatocytes, Endothelial Cells, Kupffer, Immune Cells (T cells and B cells), Stromal Cells	[13]
	mice	C57BL/6J	Male	6–11 month	HFD	PPARα, Cd36, Fabp1, Pdk4	PPARγ, FGF21, Pdk4	PPARα, Cox7a, Cox8b	PPARγ, Tnfa, IL-1b, Mcp1	PPARα	↑	↑	–	–	↑	Hepatocytes, Macrophages, Intestinal epithelial cells	[24]
	mice	C57BL/6J	Male	8 weeks	HFD	ACC, FASN, CPT1	IRS2	–	IL-6, TNF-α, IL-17, IL-10	–	↑	↑	↑	–	↑	Hepatocytes, Intestinal Epithelial Cells	[93]
	mice	C57BL/6J	Male, Female	–	HFD	Ces2a, Cyp2b10, CAR	FXR, LXRα, CAR, PXR	IRS2	Stat3, CAR, Il1r1, SAA1/SAA2 (JAK/STAT Signaling)	Ces2a, Il1r1, Saa1/Saa2	↑	–	–	–	↑	Hepatocytes, Kupffer, HSCs, Endothelial cells, Leukocytes	[15]
	mice	C57BL/6J	Male	6 weeks	HFD	PPARα, ATGL, SREBP-1c, FAS, HMGCR	FFAR2, FFAR3	PPARα	IL-6, TNF-α, FFAR2, FFAR3	SREBP-1c, FAS, HMGCR, PPARα	↑	–	↓	–	↑	Hepatocytes	[36]
	mice	C57BL/6J	Male	–	HFD	ENPP1, p-mTOR	–	–	ENPP1, TET3, p-mTOR	ENPP1, TET3, Collagen I/III, p-mTOR	↑	–	–	–	↑	Hepatocytes, HSCs	[22]
	mice	C57BL/6J	Male	8 weeks	HFD	SREBP-1c, FASN	LXRα	–	IL-1β, TNF-α, IL-6	–	↑	–	–	–	↑	Hepatocytes, Kupffer	[33]
	mice	C57BL/6J	Male, female	6 weeks	HFD	SREBP-1c, FXR signaling pathway	–	–	IL-6, FXR/SHP signaling pathway	CYP7A1, FXR/BSEP signaling pathway	↑	↑	↑	–	↑	Hepatocytes	[35]
	mice	C57BL/6J	Male	7 weeks	HFD	–	GLP-1R, GCGR	–	–	–	↑	↑	–	–	↑	–	[17]
	mice	C57BL/6J, Balb/C	Male	6–8 weeks	HFD	–	IRS1, Socs 3, HIF-1α, NF-κB	HIF-1α, NF-κB	HIF-1α, NF-κB, TGF-β	TGF-β	–	–	–	–	↑	Hepatocytes, HSCs	[29]
	rat	Sprague Dawley	Male	–	HFD	PGC-1α, CPT1A/B, PPARα	–	PGC-1α, CPT1A/B, PPARα	–	–	↑	–	↓	–	↑	Hepatocytes	[19]
	mice	C57BL/6J	Male	7 weeks	HFD	SREBP1, PPARγ, GSH, ROS/miR-155/PPARγ signaling pathway	–	SOD, GSH, ROS/miR-155/PPARγ signaling pathway	SREBP1, FAS, SCD1	–	↑	–	↓	–	–	Hepatocytes	[9]
	mice	C57BL/6J	Male	5–6 weeks	HFD	IRE1, PERK	ISR1, Akt	CHOP, IRE, p38	CHOP, JNK, p38 MAPK	–	↑	–	–	–	↑	Hepatocytes	[34]
	mice	C57BL/6J	Male	6 weeks	HFD	AMPK-SREBPs signaling pathway	INSR, AKT, GSK3β	AMPK	TNF-α, IL-6	–	↑	↑	↓	–	↑	Hepatocytes	[12]
	rat	Sprague–Dawley	Male	8–10 weeks	HFD	FAS, ACC	–	Nrf2/Keap1, TBARS, SOD, CAT	TNF-α, NF-κβ, MCP-1	–	↑	↑	–	–	↑	Hepatocytes	[30]
	mice	C57BL/6J	Male	4 weeks	HFD	Sirt1, PGC-1α, NRF1	Sirt1, FoxO3a	NRF1, TFAM	–	Sirt1	↑	–	–	–	↑	Hepatocytes	[18]
	mice	C57BL/6J	Male	8 weeks	HFD	PPARα, RXRα, AMPKα, AdipoR2	AMPKα	SIRT1	TNF-α, IL-1β	–	↑	↑	–	–	↑	Hepatocytes	[26]
	mice	C57BL/6J	Male	–	HFD	PPARγ, UNC5b	–	Netrin-1, eIF2α, CHOP	Netrin-1, UNC5b	–	↑	–	–	–	↑	Hepatocytes	[21]
	mice	C57BL/6J	Male	6 weeks	HFD	METTL14, GLS2	–	GLS2, ROS	CX3CR1/MyD88/NF-κB, DAMPs	CX3CR1/MyD88/NF-κB pathway, Col1a1, Acta2, Mmp2	↑	–	–	–	↑	Hepatocytes, Kupffer, HSCs	[32]
	mice	C57BL/6J	–	6–8 weeks	HFD	STAT3, VAV3, ABCA1, Dhcr24, Dhcr7, Cyp51	STAT3, VAV3, GLUT4, P-IRS, P-IR, P-AKT	–	STAT3, VAV3, IL-6, NF-κB, TNF-α	–	↑	↑	–	–	↑	Hepatocytes	[16]
	mice	C57BL/6J	Male	–	HFD	APN-AMPK-PPARα signaling pathway	–	APN-AMPK-PPARα signaling pathway	APN-AMPK-PPARα signaling pathway	–	↑	↑	↑	–	↑	Hepatocytes	
	mice	C57BL/6J	Male, Female	–	HFD, CD-HFD	SREBP1c, SREBP2, FASN, ACC	IRS-1, AKT, mTOR	SQSTM1	NF-κB, TNF-α, IL-6	–	↑	↑	–	–	↑	Hepatocytes	[10]
	mice	C57BL/6J	Female	10 weeks	HFD	SREBP1c, PPARα, PI3K/Akt signaling pathway	PI3K/Akt signaling pathway	ROS	TGF-β1	TGF-β1, MMP2, MMP9, TIMP1	↑	↑	–	–	↑	Hepatocytes	[14]
	mice	C57BL/6J	Male	8 weeks	HFD	PPARα/γ signaling pathways, BCKDK, PP2Cm	BCKDK, PP2Cm	–	–	–	↑	↑	↑	–	↑	Hepatocytes	[23]
CD-HFD	mice	C57BL/6J	Male	10–12 weeks	CD-HFD	PPARβ/δ, PPARα	PPARβ/δ, FGF21	–	PPARβ/δ	PPARβ/δ, NF-κB, S100A4, EMT, TGF-β, α-SMA, COL1A1	–	↑	–	–	↑	Hepatocytes	[39]
	mice	C57BL/6J	Male, Female	–	HFD, CD-HFD	SREBP1c, SREBP2, FASN, ACC	IRS-1, AKT, mTOR	SQSTM1	NF-κB, TNF-α, IL-6	–	↑	↑	–	–	↑	Hepatocytes	[10]
CDAHFD	mice	C57BL/6J	Male, Female	–	HFD, CD-HFD	SREBP1c, FASN, ACC, SCAP	IRS-1, AKT, mTOR, PKAc	SQSTM1	NF-κB, TNF-α, IL-1β, IL-6	TGF-β, α-SMA, Smad2	↑	↑	–	–	↑	Hepatocytes	[10]
	mice	C57BL/6J	Male	8 weeks	CDA‐HFD	FGF21, PNPLA3	–	–	FGF21	FGF21						Hepatocytes	[42]
	mice	C57BL/6J	Male	6–8 weeks	Amylin Liver NASH diet, CDA‐HFD	Trem2, PPARγ, Sorbs1, Plin4, DGAT2, CPT1a, Acaa1b, Ehhadh, cyp4a10/14, Aldh3a2, Cyp4a12	TLR2, IRS	GSDMD, NLRP3, IL-1β, TGF-β1	NLRP3, IL-1β, TNF-α, Socs3, TGFβ1	Trem2, OPN, Gal-3	↑	–	–	–	↑	Hepatocytes, Monocyte-derived Macrophages, MASH-associated Macrophages, Kupffer	[43]
CDAAD	mice	C57BL/6J	Male	8 weeks	CDAAD	–	–	–	IL-1β, IL-6, IL-12, HRG	α-SMA, TGF-β, MMPs, HRG	–	–	–	–	–	Hepatocytes	[44]
HFHC	mice	C57BL/6J	Male	6–8 weeks	HFHC	SREBP-1c, FASN, p300, MAPK	–	–	–	–	–	–	–	–	↑	Hepatocytes	[45]
MCD	mice	C57BL/6J	Male	6–8 weeks	Amylin Liver NASH diet, CDA‐HFD	Trem2, PPARγ, Sorbs1, Plin4, DGAT2, CPT1a, Acaa1b, Ehhadh, cyp4a10/14, Aldh3a2, Cyp4a12	TLR2, IRS	GSDMD, NLRP3, IL-1β, TGF-β1	NLRP3, IL-1β, TNF-α, Socs3, TGFβ1	Trem2, OPN, Gal-3	↑	–	–	–	↑	Hepatocytes, Monocyte-derived Macrophages, MASH-associated Macrophages, Kupffer	[43]
	mice	C57BL/6J	Male	8 weeks	HFFD, MCD	iGPX4	–	Ferritin, ACSL4, ALOX15, MDA, 4-HNE	TNF-α, IL-1β, VCAM-1	α-SMA	–	–	–	–	–	Hepatocytes, kupffer	[55]
	mice	C57BL/6J	Male	4 weeks	MCD, HFD	GLUT9/SLC2A9	GLUT9/SLC2A9	–	–	–	↑	–	–	–	–	Hepatocytes	[53]
HF-MCD	mice	Institute of Cancer Research mice	Male	8 weeks	HF-MCD	PPARγ, LXRα, FASN, CPT1A	–	HIF-1α, HO-1	NLRP3, TNF-α, IL-6, IL-10, HO-1, MCP-1	TGF-β/Smad2/3 signaling pathways, TGF-β, α-SMA, E-cadherin	↑	–	–	–	↓	Hepatocytes, Kupffer	[47]
WD	mice	C57BL/6J	Male	4 weeks	WD	CD36, FASN, LDL-R	AdipoR2	SOD, GPx, GR, FRAP	IL-10, TNF, IL-1β, IFN-γ	–	–	–	–	–	↑	Hepatocytes	[48]
	mice	C57BL/6J	Male	8 weeks	WD	p-MET	IRS1, S6K	–	–	HGF, p-MET	↑	–	–	–	↑	Hepatocytes	[50]

Currently, MASLD is recognized as a long-term injury, and there is no specific pharmacological treatment available. Although long-term medication can alleviate the development of the disease in clinical practice, it will also cause liver irritation. The researchers wondered if diet can cause MASLD, so whether a modified diet reverse the damage? If improved dietary intake can regulate MASLD development, patients may be able to avoid secondary liver damage from therapeutic drugs. Increasing evidence suggests that MASLD risk is inversely proportional to healthy dietary intake. Clinical studies have shown that the MD, a healthy dietary diet, can effectively improve MASLD symptoms in patients caused by unhealthy diet (such as WD) [65, 68–70]. Notably, in addition to being high in fat, the WD is also characterized by excessive salt content. Male patients with MASLD have significant higher daily salt intake and demonstrate a lack of behaviors associated with reduced salt consumption compared with female patients [95]. Therefore, in the daily dietary intake, patients should also pay attention to the amount of salt and not only fat intake. Given the high prevalence and complexity of MASLD caused by dietary intake, researchers have developed effective and specific treatment options based on optimal dietary patterns, are summarized in Table 2.

**Table 2:** The critical regulatory mechanism of dietary interventions on MASLD

Type of study	Dietary type	Key dietary components	Model/population	Pathological features	Key mechanisms/pathways	CMRF indicators	Other Indicators	References
Experimental	Vitamin	VC and VD3 supplementation, gut microbiota modulation, bile acid regulation	HFD-induced obese mouse model	Obesity, dyslipidemia, inflammation	Gut-liver axis regulation, bile acid metabolism, microbiota modulation	Blood pressure:reduced; HDL-c: no significant change	ALT: decreased	[79]
Experimental	Vitamin	Vitamin D3 and Omega-3 oils	Vitamin D3 supplementation, omega-3 oils	High-fat/high-fructose diet rat model	MAFLD, obesity, dyslipidemia, inflammation	Plasma triglycerides: reduced; Serum glucose: reduced; Body weight: reduced	ALT, AST, ALP: decreased; MDA, protein carbonyls: reduced	[78]
Clinical	Vitamin	4000 IU/day vitamin D	MASLD patients	Reduction in liver fibrogenic factors and liver transaminases	Involvement in VDR, fibrogenic factors, and microRNAs pathways	HDL-c: increased; Fasting glucose: reduced	ALT, AST, FBS: decreased; Laminin, Hyaluronic acid: decreased	[81]
Clinical	Fiber-enriched	6 g/12 g of Fiber-Enriched Rolls (2 times daily)	MASLD patients	Changes in SCFAs and gut microbiome diversity	SCFAs production, gut microbiome modulation	TG: not change; HDL-c:not change	ALT, AST: decreased; Gut microbiota diversity: increased; SCFAs (acetate, butyrate): increased	[84]
Clinical	Fiber-enriched	Non-free sugars, fibre, starch from whole grains, starch from refined grains	UK Biobank study participants	Variations in liver fat accumulation	De novo lipogenesis (DNL), inflammation	Refined grains: higher blood pressure, higher BMI; whole grains, fiber: lower blood pressure, lower BMI	ALT, AST: reduced with higher fiber and whole grain starch intake	[82]
Clinical	High salt intake	Daily consumption of > 6 g salt	Obese MASLD patients	Higher salt intake linked to increased cardiovascular risk	Low salt awareness and increased daily salt intake, gender differences	Hypertension, High salt intake linked to higher BMI in females	ALT, AST, GGT: slightly increased; Liver stiffness: increased	[95]
Clinical	Coffee	≥ 0 and < 1 cups/day, ≥ 1 and < 2 cups/day, ≥ 2 cups/day	KoGES Ansan_Ansung cohort, Korean participants	Advanced liver fibrosis	Suppression of fibrosis-related genes, inhibition of hepatic stellate cell activation, stimulation of Nrf2 pathway, reduction of liver inflammation	Blood pressure: reduced; Body weight: not change	FIB-4 score: lowest in ≥ 2 cups/day group; ALT, AST, r-GTP: reduced	[76]
Clinical	Dietary gluten	Gluten reduction	Coeliac Disease (CD) patients (n = 221)	Insulin resistance	Increased gut permeability, altered gut microbiota	Blood pressure: higher in MAFLD; BMI: higher in MAFLD	APRI, NFS, FIB-4 scores: higher in MASLD patients; LDL-cholesterol: higher in MASLD, PNPLA3 gene variant: increased liver fat accumulation	[58]
Experimental	Probiotics	Probiotic supplementation	Male MASLD mice model	Obesity, insulin resistance	Gut microbiota modulation, bile acid regulation	Blood glucose: reduced	ALT, AST, TG: increased	[89]
Experimental	Probiotics	Probiotic supplementation, including Lactobacillus, Bifidobacterium, and Streptococcus strains	Male MASLD mice model	hepatic steatosis, inflammation, fibrosis	Modulation of gut microbiota, reduction in hepatic lipid accumulation, inhibition of de novo lipogenesis, alteration of bile acid profile, increased SCFA production	Blood glucose: reduced	ALT, AST, TC, TG: decreased	[88]
Experimental	Probiotics	High-Fat Cholesterol Diet + A. muciniphila	Male MASLD mice model	Hepatic steatosis, inflammation	L-aspartate metabolism, lipid oxidation, bile acid metabolism, gut-liver axis interaction	Body weight: reduced	ALT, AST, ALP levels: decreased	[87]
Experimental	Intermittent fasting	Specific feeding time (8-h window)	Western diet-induced MASH model in mice	Obesity, fibrosis, hyperlipidemia	Gut microbiota rhythmic oscillations, serotonergic synapse regulation	Blood pressure:reduced; HDL: no significant change	ALT, AST, ALP: decreased; Lipid accumulation: decreased	[74]
Clinical	Intermittent fasting	Ramadan Fasting, Time-Restricted Feeding (TRF), Alternate-Day Modified Fasting, Modified Fasting Regimen	MASLD patients	Excessive liver fat accumulation	Insulin resistance, inflammation	SBP (Systolic Blood Pressure), DBP (Diastolic Blood Pressure), fasting glucose: stable	ALT, AST: decreased	[75]
Clinical	Vegetarian diet or vagetalbe or plant-based diets	Fast-foods/meats and plant-foods/prudent	Hispanic MASLD patients	More severe hepatic steatosis with fast-foods diet	Higher added sugars and fats intake	Serum glucose: increased; Blood pressure: reduced; Body weight: reduced	ALT: no significant change	[64]
Clinical	Dietary sugar restriction	Total sugar, fructose, sucrose, glucose	Latino adolescents with obesity (PNPLA3 GG genotype)	Hepatic fibrosis	Increased sugar intake leads to higher liver stiffness in GG genotype	Serum glucose: decreased	Liver stiffness: increased in GG genotype, especially with higher sugar intake	[57]
Clinical	Dietary sugar restriction	Free sugars (less than 3% of calories)	Adolescent boys with biopsy-proven MASLD	Insulin resistance	Reduced hepatic de novo lipogenesis (DNL), reduced insulin-mediated lipogenesis	Plasma triglycerides: reduced; Serum glucose: reduced; Body weight: reduced	ALT: decreased; Hepatic fat: decreased	[59]
Clinical	Dietary sugar restriction	Less than 10% of calories from added sugars	Children with biopsy-proven MASLD (n = 119)	MASH, hepatic fibrosis	Reduced added sugar intake associated with less severe liver steatosis	Plasma triglycerides: reduced; HDL-c: incerased	ALT: stable; Hepatic steatosis: decreased	[71]
Clinical	Dietary sugar restriction	Higher intake of alcohol, sugar, red meat, and pastries	MASLD patients with harmful alcohol intake	Advanced fibrosis, severe steatosis	Increased inflammation due to alcohol and diet	-	ALT, AST, GGT: elevated; CAP: increased; Liver stiffness: increased	[56]
Experimental	Glucan or oat	Oat beta-glucan supplementation	Male C57BL/6 mice (WSD)	Inflammation, hepatic fibrosis	Modulation of gut microbiota, TLR signaling reduction	Serum glucose: not change	ALT, AST, GLDH: decreased, Collagen: decreased	[85]
Experimental	Ketogenic diet	High fat, low carbohydrate, low protein	Western diet-induced mouse model	Hepatic insulin resistance	IL-6-JNK signaling activation	Serum glucose: increased	ALT, AST: increased	[60]
Clinical	Ketogenic diet	20–50 g carbs/day, 800 kcal/day, high protein	Overweight/obese subjects with MASLD (n = 87)	Low-grade inflammation, fibrosis	Reduced carbohydrate intake, ketosis induced	Plasma triglycerides reduced; Serum glucose reduced; Plasma HDL-cholesterol: Blood pressure reduced (both systolic and diastolic); Body weight reduced	Liver stiffness: decreased; ALT: decreased	[61]
Clinical	Ketogenic diet	Low-calorie intake (< 800 kcal/day), very low carbohydrates	Overweight and obese patients with MASLD	Hepatic steatosis, fibrosis, obesity	Reduction of insulin resistance, hormonal effects	Plasma triglycerides Higher baseline in men; greater reduction in men; Serum glucose More significant reduction in men; Plasma HDL-cholesterol Higher baseline in women; greater reduced in women; Blood pressure: Greater systolic BP reduction in men; similar diastolic BP reduction; Body weight Greater waist circumference reduction in men	ALT, AST, γGT: decreased; CAP: decreased	[63]
Clinical	Ketogenic diet	Very lowcalorie intake (< 800 kcal/day), low carbohydrates	Overweight and obese patients with MASLD	Hepatic steatosis, fibrosis, obesity	Ketogenesis, reduction of insulin resistance, anti-inflammatory effects	Plasma triglycerides: reduced; Serum glucose: reduced; Plasma HDL-c: reduced; Blood pressure: reduced (both systolic and diastolic); Body weight: reduced	ALT, γGT: decreased; CAP: decreased	[62]
Experimental	Mexican diet	Opuntia ficus indica (nopal), Theobroma cacao (cocoa), Acheta domesticus (crickets)	MASLD mice and overweight/obese patients	Obesity, gut dysbiosis	Increased SCFAs, downregulation of TNFα, upregulation of antioxidant enzymes	Body weight: reduced	ALT, SCFAs: decreased	[86]
Clinical	Mediterranean diet	High in vegetables, fruits, legumes, nuts, olive oil	Korean population (KoGES study)	Obesity, metabolic risk	Gene-diet interaction involving GCKR rs780094	Plasma triglycerides: reduced; Serum glucose: reduced; Plasma HDL-c: increased; Blood pressure: reduced; Body weight: reduced	ALT, AST: decraesed	[68]
Clinical	Mediterranean diet	High-quality carbohydrates, Fresh fruits and vegetables, Legumes and nuts	Adults with MASLD (n = 228 cases, 228 controls)	Fibrosis, inflammation	Increased intake of vegetables, whole grains, lower sugar and unhealthy fats	Plasma triglycerides: increased; Serum glucose: increased; Plasma HDL-c: reduced; Blood pressure: increased; Body weight: increased	ALT: stable; Fibrosis severity: decreased; Whole grains and vegetable score associated with reduced MASLD risk	[70]
Clinical	Mediterranean diet	Mediterranean-based, high antioxidants, polyunsaturated fats	MASLD patients (n = 98), control (n = 45)	Obesity, liver steatosis, inflammation	Mediterranean adherence, antioxidant intake	Plasma triglycerides: reduced; Serum glucose: reduced; Plasma HDL-cholesterol: increased; Body weight: reduced	Leptin: decreased; M65: significantly reduced	[65]
Clinical	Mediterranean diet	5:2 Diet, high plant-based foods, olive oil, fish	Diverse participant group (aged 35–69)	Visceral adiposity, liver fat	Energy restriction, ketosis, improved insulin sensitivity	Plasma triglycerides: reduced; Serum glucose: reduced; Blood pressure: reduced	ALT, AST: decreased; liver fat content: decreased	[72]
Clinical	Mediterranean diet	High in fruits, vegetables, whole grains, nuts, olive oil	Veterans in primary care with MAFLD	Obesity, MAFLD, fibrosis	Anti-inflammatory, improved insulin sensitivity	Body weight: reduced	ALT, AST: elevated; Liver stiffness: increased; CAP: increased	[69]

Attentively, the findings from animal experiments and organoid studies not only provide crucial insights into the pathogenesis of MASLD but also help guide dietary interventions for improving the condition in patients. Recently, one study demonstrated that controlling carbohydrate intake, particularly by reducing high glycemic index (GI) carbohydrates, could significantly alleviate liver burden and enhance metabolic health. In animal models, a hypercaloric low-carbohydrate, high-fat diet has been shown to protect against MASLD by enhancing hepatic fatty acid oxidation, suppressing DNL, and improving insulin sensitivity [96]. This mechanistic understanding was further supported by clinical evidence, where a study in humans found that restricting dietary sugars led to 10% reduction in hepatic DNL, 7% decrease in liver fat, and significant improvements in insulin sensitivity, fasting glucose levels and triglyceride levels [59]. These clinical improvements were likely related to reduced hepatic lipid accumulation and improved metabolic control. Animal models have identified enhanced fatty acid oxidation and suppressed DNL as key mechanisms. These findings directly inform the selection of clinical biomarkers, such as hepatic DNL, and dietary intervention targets, including carbohydrate restriction, thereby providing a foundation for developing precise nutritional strategies for the management of patients with MASLD. Mindfully, IF may be better suited to KD/VLCDK dietary therapy or the ATI-free diet to combat the burden of glucose metabolism. Besides, low-carbohydrate diets (LCDs) also exhibited dual effects on MASLD, offering short-term metabolic benefits that may be counterbalanced by long-term hepatic risks. Acute interventions with LCDs have been shown to enhance hepatic fat oxidation, reduce systemic insulin levels and promote weight loss through ketogenesis [60]. For the general obese MASLD patients, an IF diet pattern can painlessly help patients in slowing the progression of HDL-C, blood pressure, IR and lipid profiles in an even better fashion. However, prolonged carbohydrate restriction triggered hepatic insulin resistance via IL-6-JNK pathway activation, impaired insulin-mediated suppression of gluconeogenesis, and elevated non-esterified fatty acid influx from adipose tissue lipolysis, collectively exacerbating hepatic lipid accumulation. Concurrent gut microbiota dysbiosis characterized by reduced butyrate/propionate producing bacteria, elevated *Bacteroidetes/Firmicutes* ratio, and altered BA metabolism, and disrupts gut-liver crosstalk. While initially boosting lipid oxidation, chronic LCD adherence suppressed glycolysis-mediated ATP production, induced mitochondrial oxidative stress, and upregulated fibrosis-related genes (Col1a1, α-SMA, TGF-β), potentially accelerating fibrogenesis [60, 97, 98].

For MASLD patients with abnormal fat metabolism and prominent inflammatory phenotype, nutritional supplements such as prebiotics and trace elements (vitamin, dietary fiber, antioxidants) can be considered to stimulate related metabolic signaling pathways through the hepato-intestinal axis, consequently alleviating MASLD. Peculiarly, studies have indicated that dietary supplements Omega-3 (including EPA and DHA) was a polyunsaturated fatty acid (PUFAs) that had significant anti-inflammatory effects [99, 100]. Specifically, it suppressed pro-inflammatory M1 markers (TNF-α/IL-6) and enhanced anti-inflammatory M2 markers (CD206⁺/Arg1⁺) though PPARγ and Kruppel-like factor 4 pathways in both Horsfall Protein-1 cells and MASLD models [101]. This anti-inflammatory effect was mediated by specialized pro-resolving mediators (SPMs) derived from DHA/EPA: maresin-1 (from DHA) promoted Kupffer cell M2 polarization to alleviate liver fibrosis, while resolvin D1 (from EPA) enhanced efferocytosis of apoptotic. Clinical studies further showed that omega-3 supplementation lowered triglyceride levels and reduced macrophage infiltration in MASLD patients [102]. While Omega-3 fatty acids ameliorated MASLD by resolving inflammation and fibrosis through metabolic reprogramming of immune cells, the persistent dysregulation of immune checkpoints such as programmed cell death protein 1 (PD-1) in exhausted T cells suggested a potential synergistic therapeutic strategy to together target metabolic dysfunction and immune exhaustion in advanced MASLD. In MASLD mice model, chronic lipid accumulation and inflammation drove PD-1 overexpression, particularly on cytotoxic CD8^+^ T cells, leading to T cell exhaustion characterized by impaired cytokine production, proliferative and cytolytic abilities. Concurrently, PD-1 signaling facilitated immune evasion of damaged hepatocytes and pre-malignant cells by enhancing Treg cell activity and suppressing effector T cell responses [103].

Current studies have pointed out that in animal models, probiotic supplementation can effectively improve the balance of intestinal microbiota and promote SCFAs (microbial metabolites) to activate Nrf2 or AMPK signaling pathway, or inhibit the signal transduction of PPAR-γ and inflammatory factors (IL-17, TNF-α and IL-6), contributing to the recovery of liver metabolic disorders [83–89, 92, 93]. As a pivotal factor in the development of obesity and gut microbiota disorders, SCFAs exhibit immunomodulatory and anti-inflammatory properties that contribute to the maintenance of gut barrier homeostasis. Besides SCFAs, BAs can be produced by the gut microbiota and influence the intestinal flora balance. Studies have shown that UDCA and LCA (secondary metabolites of BAs) activated G-protein-coupled bile acid receptor (TGR5) to boost fat burning in brown fat tissue [104]. Imbalanced of gut bacteria (*Firmicutes* outnumbered *Bacteroidetes*) caused by unhealthy diet led to two key problems arose. Firstly, the imbalance increased energy absorption and fat storage, directly contributing to liver fat buildup. Secondly, it reduced the production of UDCA and LCA, which were critical for activating TGR5 and maintaining metabolic health. Lower levels of these BAs slowed down fat burning in brown fat, worsened metabolic dysfunction, and accelerated liver disease progression. This dual disruption of energy balance and BA signaling highlighted how gut microbiota interactions directly drove MASLD progression [105].

Given the variability in the presentations of MASLD among individuals, it is essential to implement tailored treatment approaches. However, these specific dietary strategies necessitate further validation through basic research and targeted clinical studies.

## Conclusion

Preventive healthcare has consistently been emphasized as crucial; in fact, a healthy diet can not only improve the progression of MASLD but also reduce the incidence of the disease. It is essential for everyone to establish a healthy diet and adhere to the principle that 'prevention is better than cure.'

## Abbreviations

ACC: Acetyl-CoA carboxylase
AMPK: AMP-activated protein kinase
BA: Bile acid
CDAAD: Choline-deficient L-amino acid diet
CDAHFD: Choline-deficient, L-amino acid-defined, high-fat diet
CD-HFD: Choline-deficient high-fat diet
FA: Fatty acid
FAS: Fatty acid synthase
FXR: Farnesoid X receptor
HFD: High-fat diet
HFHCD: High-fat high-cholesterol diet
HF-MCD: High-fat methionine- and choline-deficient diet
IF: Intermittent fasting
IR: Insulin resistance
KD: Ketogenic diet
LDL-R: Low density lipoprotein receptor
MAFLD: Metabolic dysfunction-associated fatty liver disease
MASH: Metabolic dysfunction-associated steatohepatitis
MCD: Methionine-choline deficiency diet
MD: Mediterranean diet
PA: Palmitate
PPARα: Peroxisome proliferator-activated receptor alpha
SCFA: Short-chain fatty acid
SREBP: Sterol regulatory element-binding protein
TG: Triglycerides
TGF-β1: Transforming growth factor
WD: Western diet
